# Off‐label prescribing of immune checkpoint inhibitor therapy at a single pediatric cancer center

**DOI:** 10.1002/cam4.7154

**Published:** 2024-04-17

**Authors:** Ajami Gikandi, Susan N. Chi, Kee Kiat Yeo, Allison F. O'Neill, David S. Shulman, Steven G. DuBois, Natalie B. Collins

**Affiliations:** ^1^ Harvard Medical School Boston Massachusetts USA; ^2^ Dana‐Farber/Boston Children's Cancer and Blood Disorders Center Harvard Medical School Boston Massachusetts USA

**Keywords:** experimental therapeutics, immune checkpoint, off‐label, pediatric cancer

## Abstract

**Background:**

Immune checkpoint inhibitors (ICI) have improved outcomes in a variety of adult cancers and are prescribed with increasing frequency across oncology. However, patterns of off‐label use of ICI in pediatrics remain unclear.

**Methods:**

This is a single‐institution, retrospective cohort study evaluating off‐label ICI use in pediatric and young adult patients with cancer treated at our institution from 2014 to 2022. Response was based on clinician assessment derived from clinical records. Immune‐related adverse events (iRAEs) were classified according to CTCAE v5.0.

**Results:**

We identified 50 unique patients treated with off‐label ICI (28 with solid tumors, 20 with central nervous system (CNS) tumors, 2 with hematologic malignancies). At time of ICI initiation, only five patients (10%) had localized disease, and all but one patient was treated in the relapsed/refractory setting. All patients were treated with the FDA‐approved weight‐based dosing recommendations. Overall, there was disease control in 21 patients (42%), with best response including one complete response (melanoma), two partial responses (high‐grade glioma, CNS nongerminomatous germ cell tumor), and 18 patients with stable disease. Forty‐four patients (88%) eventually experienced disease progression. Among 22 patients (44%) experiencing iRAEs, 10 (20%) had a grade ≥3 irAE, 12 (24%) required corticosteroids, and 14 (28%) required ICI discontinuation. irAE occurrence was associated with significantly improved progression‐free survival (HR 0.35; 95% CI: 0.18 to 0.68; *p* = 0.002) and overall survival (HR 0.33; 95% CI: 0.17 to 0.66; *p* = 0.002).

**Conclusions:**

At our institution, ICI was most commonly prescribed in the relapsed/refractory setting to patients with metastatic disease. The treatment was generally well‐tolerated in the pediatric population. The overall response rate was low, and the majority of patients eventually experienced disease progression. A few patients, however, had durable treatment responses. Further studies are needed to identify which pediatric patients are most likely to benefit from ICI.

## Graphical abstract

Patterns of off‐label use of immune checkpoint inhibitors (ICI) in pediatric and young adult patients with cancer remain unclear. At our institution, we found that off‐label ICI was most commonly prescribed in the relapsed/refractory setting to patients with metastatic disease, and the treatment was generally well‐tolerated. The overall response rate was low, and the majority of patients eventually experienced disease progression. A few patients, however, had durable treatment responses.

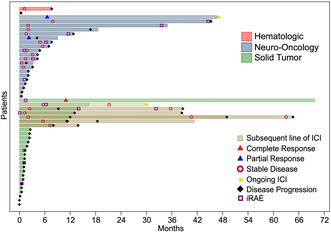

## 1 INTRODUCTION

Major barriers exist in pediatric cancer drug development.^1^, ^2^ As a result, commercially available drugs are often used for indications other than those approved for use. This off‐label use may involve a different age group, therapeutic indication, or dosing strategy than specified on the regulatory agency approved label.^3^ There are many potential benefits to off‐label prescribing, especially in rare diseases with few treatment options, such as pediatric cancers.^4^ Despite several legislative efforts over the last few decades, the number of drugs approved for pediatric oncology indications continues to lag behind the number approved for adult indications.^5^, ^6^, ^7^ Consequently, many oncology drugs used in the pediatric setting are prescribed off‐label. Although prevalent, few studies have described patterns in off‐label prescribing of anticancer agents in pediatric oncology. This gap in the literature may be due to publication bias favoring studies with positive results, negative connotations associated with the term “off‐label,” or concerns regarding administration of drugs not federally approved for children. One study evaluating the off‐label use of targeted therapy in pediatric cancers from 2007 to 2017 observed an overall prevalence of 9.2%.^8^ In an Italian single‐center study describing 45 patients treated with off‐label or compassionate use of targeted anticancer therapies, the majority of use was in relapsed or refractory disease, 71% experienced at least one adverse drug reaction, and 52% achieved a complete response.^9^

Over the last decade, immune checkpoint inhibition (ICI) has revolutionized the field of medical oncology, and the number of patients with adult malignancies treated with ICI has dramatically increased.^10^, ^11^ Retrospective studies and early phase clinical trials have demonstrated ICI to be safe and tolerable in pediatric patients with a variety of malignancies,^12^, ^13^, ^14^, ^15^, ^16^, ^17^, ^18^, ^19^, ^20^, ^21^, ^22^, ^23^ including central nervous system tumors.^24^, ^25^ ICI has, however, only been approved for a select group of pediatric cancers.^26^ Early phase clinical trials of ICI agents have largely been disappointing in pediatrics, at least partially attributed to lower immunogenicity due to low neoantigen burden.^12^, ^27^ Similar to off‐label drug use in children in general, few studies describe off‐label prescribing of ICI. One study analyzing outcomes of 16 adult patients with advanced cancers treated with off‐label drugs based on next‐generation sequencing results observed stable disease at 16 weeks in all patients treated with pembrolizumab or nivolumab for tumors with PD‐L1 expression or *TP53* mutations.^28^ Another study used an online database to describe patterns of off‐label use in 527 adults.^29^ Previously, our group described patterns of off‐label use in pediatric patients with central nervous system (CNS) diagnoses.^24^

To further clarify patterns of off‐label ICI use in pediatric patients, we conducted a single‐center, retrospective study of children and young adults with cancer prescribed off‐label ICI at our institution. The goal of this study was to describe the prevalence, prescribing patterns, agents, and dosing strategies of off‐label ICI. In addition, we also aimed to summarize the demographic and clinical characteristics of patients treated with off‐label therapy, as well as the effectiveness and toxicity of off‐label ICI.

## 2 METHODS

### 2.1 Ethical approval

This study was approved by Dana‐Farber/Harvard Cancer Center Institutional Review Board (Protocol no. 18‐229) with a waiver of informed consent.

### 2.2 Design and patient population

We used multiple institutional databases to identify pediatric patients who received off‐label ICI for an oncologic indication at Dana‐Farber/Boston Children's Cancer and Blood Disorders Center between January 1, 2007 (the first year the current electronic medical record was used) and March 25, 2022. ICI included drugs targeting PD‐1, PD‐L1, or CTLA‐4. Off‐label use was defined as a patient being prescribed a commercially available drug that is not FDA approved for the oncologic indication in pediatric patients at time of first dose. FDA‐approved indications and drug approval dates were obtained from https://www.access.fda.gov/. Inclusion criteria included any patient who received off‐label ICI according to this definition. Exclusion criteria included patients who received ICI treatment as part of a clinical trial or compassionate access protocol. Six patients with fibrolamellar carcinoma were also excluded since they are the focus of a concurrent study from our institution.

### 2.3 Variables

We performed a detailed review of the electronic health record to capture additional information on patients who received off‐label ICI. We collected information on sex, disease category, cancer diagnosis, stage at diagnosis, prior therapy, immunohistochemistry (IHC) staining for PD‐L1 and/or PD‐1, and tumor mutational burden (TMB) testing by OncoPanel.^30^ For each episode of off‐label use, we collected information on stage at treatment, treatment setting (frontline versus relapsed/refractory), dosing scheme, duration of therapy, best overall response, duration of best response, immune‐related adverse events (irAEs), and reasons for stopping therapy. Duration of off‐label therapy was defined as days from first dose to last dose prior to discontinuation, or the date of an interruption causing the next planned dose of ICI to be delayed >4 weeks. Delays ≤4 weeks for management of suspected irAE did not count as an interruption. Patients who only received one dose of ICI had zero days as their duration of off‐label therapy. The first line of ICI was considered to have ended when patients received an ICI not included in their original regimen or when all ICI agents in the original regimen were discontinued for 60 days.

### 2.4 Response assessment

Response was based on treating clinician assessment derived from review of medical records in accordance with prior studies.^8^, ^31^ Primary imaging studies were not reviewed to verify response status because objective assessment with RECIST,^32^ iRECIST,^33^ and iRANO^34^ was beyond the scope of this retrospective review. Best overall response was classified as progressive disease (PD), stable disease (SD), partial response (PR), or complete response (CR). Disease control rate (DCR) was defined as the proportion of patients in whom the best response was a CR, PR, or SD. Overall response rate was defined as the proportion of patients in whom the best response was a CR or PR. When there were equivocal findings of progression at an assessment, this assessment was classified as progressive disease if the subsequent assessment confirmed progression. Duration of best response was defined as the number of days from the assessment that showed the best response to the next assessment with progression. If there was disease control at the time of ICI discontinuation or interruption, the duration of best response was continued until the next assessment showing progression, or the most recent assessment prior to a new line of therapy being started, if disease control remained the same. Patients who were coded as having progressive disease as best response had progressive disease on first response assessment. Patients with progressive disease as best response were coded as having a duration of best response of zero days. Cancer type, including the natural history of disease, concomitant therapy, and duration of response were not taken into account when determining response status. Response assessments were only taken into account for patients with measurable disease.

### 2.5 Toxicity assessment

iRAEs were classified according to Common Terminology Criteria for Adverse Events (CTCAE) version 5.0 by reviewer interpretation. Information on whether irAEs required corticosteroid treatment was also captured. Clinically meaningful toxicity was defined as any unplanned clinic visits, emergency department visits, or unplanned hospital admissions at least possibly related to off‐label therapy, regardless of iRAE grade.

### 2.6 Statistical analysis

Statistical analysis was performed using STATA software, version 17 (StataCorp, College Station, TX). Descriptive statistics (frequencies and proportions) were used to summarize demographic and clinical characteristics of the patient cohort. Stratified data were compared using the Kruskal–Wallis or paired t‐tests for continuous variables and the chi‐squared test for categorical variables. Multivariate logistic regression using a priori defined variables (disease category, ICI regimen) were used to identify predictors of disease response. Estimates of progression‐free survival (PFS) and overall survival (OS) obtained with the Kaplan–Meier method were compared using the log‐rank test. Univariate Cox proportional hazard regression models were used to determine hazard ratios. Statistical significance was defined at *p* < 0.05.

## 3 RESULTS

### 3.1 Characteristics of patients treated with ICI

During the time period assessed, we identified 50 patients who received off‐label ICI prescriptions and satisfied our inclusion/exclusion criteria. The demographic and clinical characteristics of these patients are described in Table 1. Specific cancer diagnoses are listed in Table 2. The median (range) age at initiation of first‐line ICI was 14.5 (3.0–36.0) years. Of the patients with solid tumors (*n* = 28), the most common diagnoses were Ewing sarcoma (*n* = 4) and alveolar rhabdomyosarcoma (*n* = 3). High‐grade gliomas (*n* = 12) were the most common neuro‐oncology (*n* = 20) diagnoses, with the most common subtype being diffuse midline glioma (*n* = 4). We previously described patterns of off‐label ICI use in 11 of these neuro‐oncology patients.^24^ The two cases of hematologic malignancy included one patient with therapy‐related myelodysplastic syndrome (MDS) that developed after allogeneic stem cell transplant for acute lymphoblastic leukemia (ALL), and another patient with isolated central nervous system (CNS) acute myeloid leukemia (AML) that developed from B‐ALL that underwent a lineage switch. At the time of ICI initiation, all but one patient received treatment in the relapsed or refractory setting, and only 5 (10%) had localized disease. No patients in disease remission received ICI. The patient who received frontline ICI had metastatic clear cell sarcoma that was negative for PD‐L1 on IHC with no information on TMB. They received two cycles of pembrolizumab prior to discontinuation due to disease progression. Radiation (*n* = 17, 34%) and molecular targeted therapy (*n* = 17, 34%) were the most recent treatment modalities preceding ICI, and 26 patients (52%) had previously received more than one line of chemotherapy. Twenty‐six patients (52%) received concomitant therapy (surgery, chemotherapy, other targeted therapy, radiation, other) alongside ICI, with radiation (*n* = 17) being the most common co‐delivered therapy. TMB data prior to ICI were available on 24 patients with a mean TMB of 6.5 mutations/Mb and a median (range) TMB of 5.3 (1.5–30.4) mutations/Mb. As part of routine clinical care, 10 patients underwent IHC staining with PD‐1 and/or PD‐L1.

**TABLE 1:** Patient characteristics (*N* = 50).

Variable	*n*
Median age at off‐label ICI, years (range)	14.5 (3.0–36.0)
Female	17 (34%)
Disease category	
Hematologic malignancy	2 (4%)
Solid tumor	28 (56%)
Neuro‐oncology	20 (40%)
Stage at diagnosis^a^	
Localized	22 (46%)
Regional	9 (35%)
Metastatic	17 (35%)
Stage at first off‐label ICI^a^	
Localized	5 (10%)
Regional	7 (15%)
Metastatic	36 (75%)
IHC staining for PD‐L1 and/or PD‐1	10 (20%)
TMB measured	24 (48%)
Frontline ICI	1 (2%)
Prior therapy	
Chemotherapy	34 (68%)
Radiation therapy	39 (78%)
Molecular targeted therapy	31 (62%)
Surgery	34 (68%)
Stem cell transplant	3 (6%)
Other	8 (16%)
Cryoablation	2
Interferon alpha 2b	3
Adoptive T‐cell transfer	1
^131^I‐MIBG	1
Cannabinoid and THC oil	1

*Note*: Data presented as *n*/*N* (%) unless otherwise noted.

Abbreviations: ICI, immune checkpoint inhibition; IHC, immunohistochemistry; MIBG, metaiodobenzylguanidine; PD‐1, programmed cell death protein 1; PD‐L1, programmed death ligand 1; THC, tetrahydrocannabinol; TMB, tumor mutational burden.

^a^ Only includes patients with non‐hematologic cancers (*n* = 48).

**TABLE 2:** Cancer diagnoses of patients treated with ICI.

	*n*
Solid tumor (*n* = 28)	
Alveolar rhabdomyosarcoma	3
Anaplastic chordoma	1
BCOR‐CCNB3 sarcoma	1
Clear cell sarcoma	1
CMMRD‐associated small intestine adenocarcinoma	1
Desmoplastic small round cell tumor	1
Diffuse anaplastic Wilms tumor	1
Embryonal rhabdomyosarcoma	1
Epithelioid sarcoma	2
Ewing sarcoma	4
Hepatocellular carcinoma	2
Melanoma	1
Metastatic carcinoma of unknown primary	1
Neuroblastoma	1
Osteosarcoma	2
Perivascular epithelioid cell neoplasm	1
Sclerosing epithelioid fibrosarcoma	1
Synovial sarcoma	1
Transitional liver cell tumor	1
Wilm's tumor	1
Neuro‐oncology (*n* = 20)	
High‐grade glioma	5
Diffuse midline glioma	4
Radiation‐induced high‐grade glioma	2
Anaplastic oligodendroglioma	1
Anaplastic ependymoma	1
High‐grade neuroepithelial tumor	1
Medulloblastoma	1
Pineoblastoma	1
Atypical teratoid rhabdoid tumor	1
Embryonal tumor with multilayered rosettes	1
Adamantinomatous craniopharyngioma	1
Nongerminomatous germ cell tumor	1
Hematologic (*n* = 2)	
Isolated central nervous system acute myeloid leukemia	1
Therapy‐related myelodysplastic syndrome	1

### 3.2 Patterns of off‐label ICI over time

**FIGURE 1:**
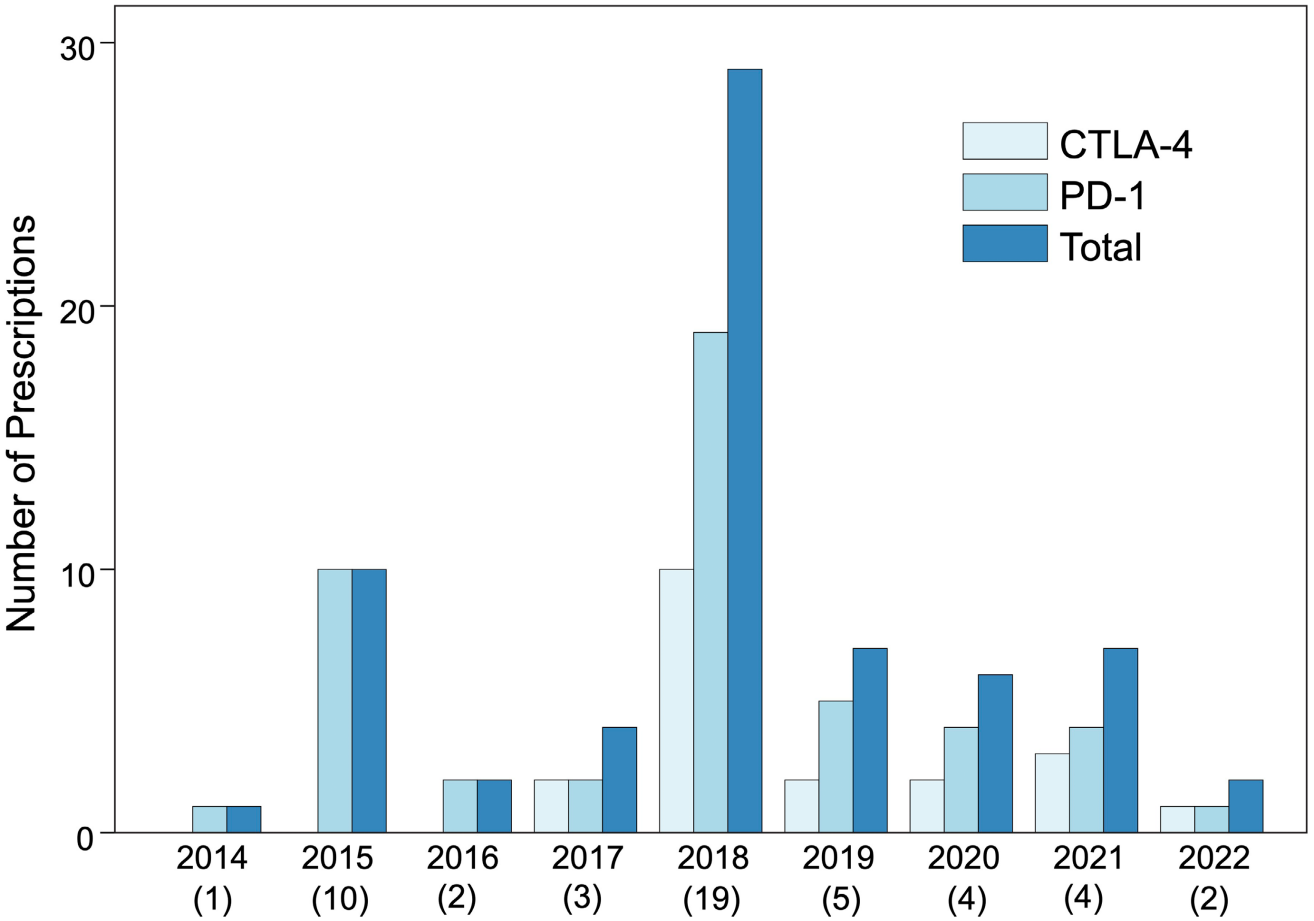
Number of prescriptions for each immune checkpoint inhibitor (ICI) class by year of treatment initiation. Number in parenthesis is the number of unique patients who initiated ICI that year (*n* = 50). Component drugs of dual ICI (e.g., combination CTLA‐4 and PD‐1) were counted separately. CTLA‐4 (ipilimumab), PD‐1 (nivolumab, pembrolizumab). *CTLA‐4*, cytotoxic T‐lymphocyte‐associated protein 4; *PD‐1*, programmed cell death protein 1.

Off‐label ICI prescribing increased from the first prescription in 2014 to a peak in 2018 followed by a decrease in off‐label prescribing (Figure 1). Of 50 off‐label prescriptions, 30 (60%) were anti‐PD‐1 monotherapy, 18 (36%) were combination anti‐CTLA‐4/PD‐1, and 2 (4%) were anti‐CTLA‐4 monotherapy. The specific ICI regimens were single‐agent pembrolizumab (*n* = 22), nivolumab/ipilimumab (*n* = 18), single‐agent nivolumab (*n* = 8), and single‐agent ipilimumab (*n* = 2). Both prescriptions for ipilimumab monotherapy were for patients with hematologic malignancies (therapy‐related MDS and isolated CNS AML). Among six patients who received more than one line of ICI, first‐line ICI was stopped due to disease progression and iRAEs in three and three patients, respectively.

### 3.3 Dosing of off‐label ICI

Single‐agent pembrolizumab (2 mg/kg to a maximum 200 mg flat dose every 3 weeks), nivolumab/ipilimumab (3 mg/kg nivolumab and 1 mg/kg ipilimumab every 3 weeks), single‐agent ipilimumab (up to 10 mg/kg every 3 weeks), and single‐agent nivolumab (3 mg/kg every 2 weeks) were all prescribed at FDA‐approved doses in adults. Pembrolizumab was the only ICI regimen modified. Two patients had pembrolizumab dosing modified from 2 mg/kg every 3 weeks to 4 mg/kg every 6 weeks, which was an FDA‐approved dosing regimen across adult indications, while another patient had their dose temporarily decreased from 2 to 1 mg/kg when treated concurrently with a cancer vaccine.

### 3.4 Duration of off‐label ICI

**FIGURE 2:**
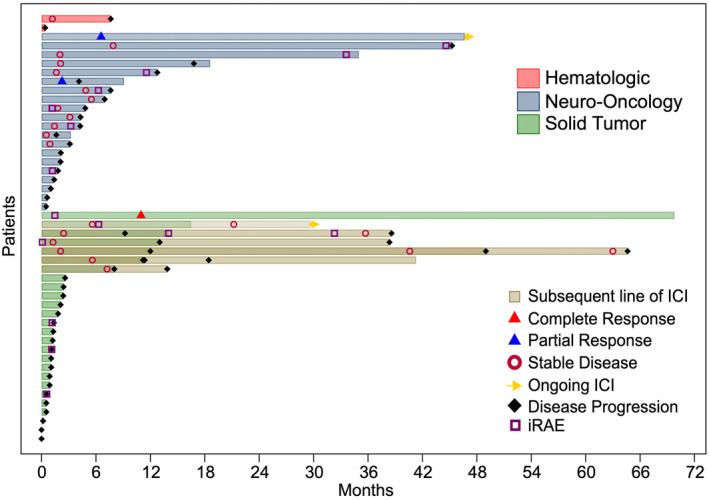
Duration of best response to immune checkpoint inhibition (ICI). Best response was based on clinician assessment. Each bar represents a patient who had disease response assessed at least once (*n* = 48). Mixed color bars represent patients who received two lines of ICI with the first bar representing first‐line ICI and the second bar representing second‐line ICI. Immune‐related adverse events (irAEs) that met toxicity criteria are defined as unplanned clinic visits, emergency department visits, or unplanned hospital admissions that were at least possibly related to off‐label therapy.

The median (IQR) duration of ICI therapy was 43 (18–189) days with a broad range of 0–1400 days (Figure 2). Of the eight patients who had a duration of ICI therapy of zero days due to receipt of only one dose of ICI, six stopped ICI due to disease progression, whereas the remaining two died from disease progression prior to the second dose of ICI. Median (IQR) duration of ICI in patients with solid tumors was shorter than in patients with hematologic and neuro‐oncology malignancies (21 [6–69] days vs. 105 [36–273] days, *p* = 0.014). The most common reasons for ICI cessation were disease progression and irAEs, with 33 (66%) patients stopping ICI due to disease progression alone, 7 (14%) stopping due to irAEs alone, and 7 (14%) patients stopping due to both disease progression and irAE. At the time of data cutoff for this analysis, two patients were continuing to receive off‐label ICI. One patient with a CNS nongerminomatous germ cell tumor has continuously received more than 75 doses of single‐agent nivolumab after initially receiving 4 cycles of nivolumab/ipilimumab. Another patient with constitutional mismatch repair deficiency (CMMRD)‐related small intestine cancer has received more than 25 cycles of pembrolizumab, though this was intermittent due to lack of detectable disease at times.

### 3.5 Response assessment

**FIGURE 3:**
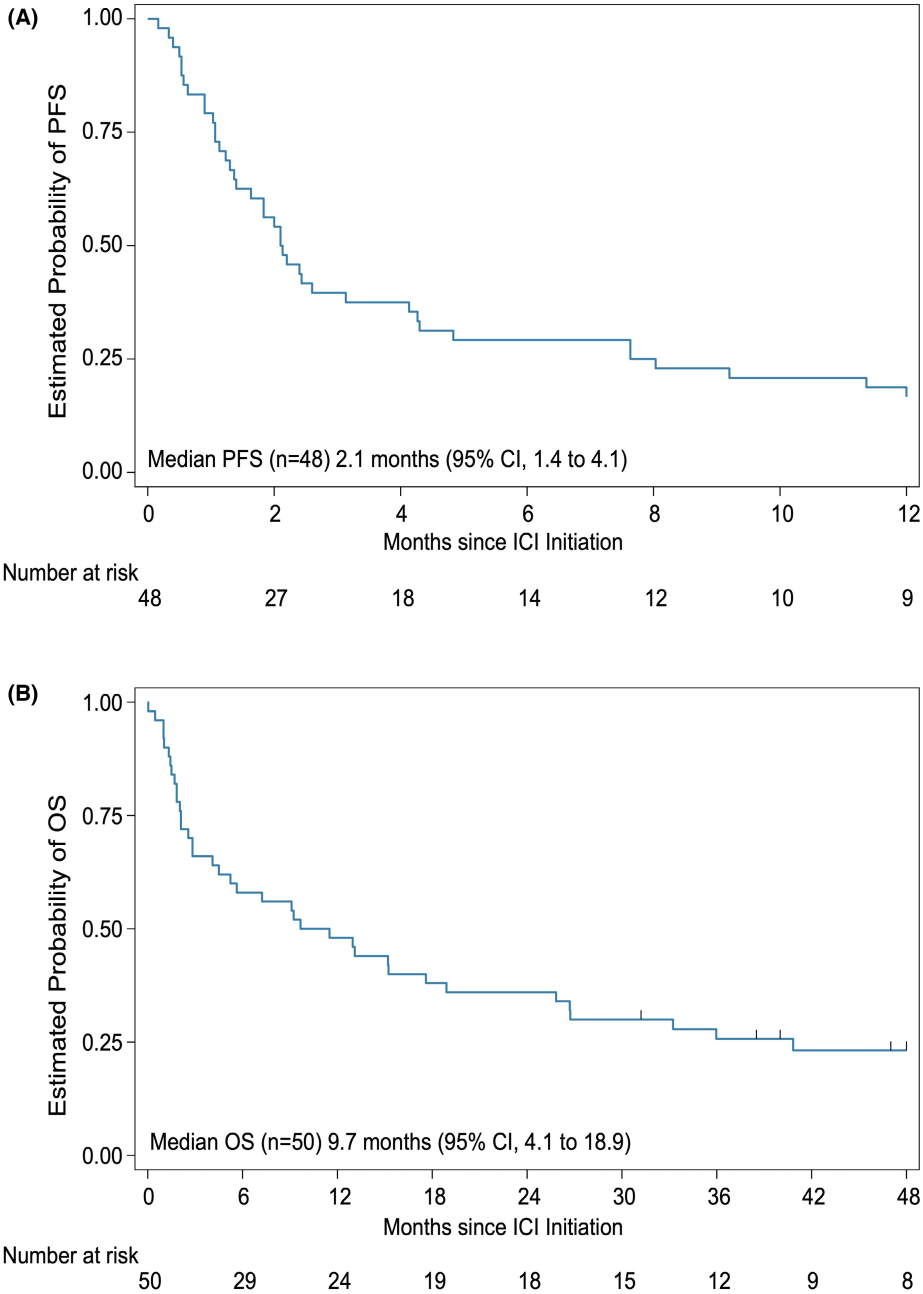
(A) Estimated progression‐free survival (PFS) of 48 patients who had disease response assessed. (B) Estimated overall survival (OS) of all 50 patients in cohort.

Clinician assessment was derived from the medical record in 48 patients. Two patients died before response status could be evaluation. Clinician assessment of response status was most often based on radiologic response. Patients with hepatocellular carcinoma also had response assessed by trends in alpha‐fetoprotein level. The patient with therapy‐related MDS had response assessed by absolute neutrophil count and transfusion‐dependence. One patient (2%) had a complete response (melanoma), two patients (4%) had a partial response (high‐grade glioma and CNS nongerminomatous germ cell tumor), and 18 patients (36%) had stable disease, resulting in an overall response rate of 6% and a disease control rate of 42% (Table 3). Disease control rates were 65% (13/20) in neuro‐oncology diagnoses, 37% (7/19) in solid tumor diagnoses, and 50% (1/2) in hematologic diagnoses. In patients with stable disease, the median (IQR) duration of control was 6.4 (2.8–11.8) months. In the six patients who received multiple lines of ICI, the best response was always during the first line of ICI therapy, and the median (IQR) duration of first‐line and second‐line ICI were 257 (189–360) days and 95 (32–253) days, respectively. Overall, clinician assessment based on clinical notes described disease progression in 44/48 patients (92%) who had disease response assessed. PFS was determined for all 48 patients who had disease response assessed, whereas OS was determined for all 50 patients. The median estimated PFS and OS of the entire cohort were 2.1 (95% CI, 1.4 to 4.1) months and 9.7 (95% CI, 4.1 to 18.9) months, respectively (Figure 3). Estimated PFS (hazard ratio 0.73; 95% CI, 0.40 to 1.34; *p* = 0.31) and OS (hazard ratio 0.58; 95% CI, 0.30 to 1.10; *p* = 0.09) did not differ significantly between solid tumors and other disease categories.

**TABLE 3:** Response and toxicity by disease category.

	Hematologic *N* = 2	Solid tumor *N* = 28	Neuro‐oncology *N* = 20
Duration of ICI, days	73 (0–145)	21 (6–70)	112 (42–383)
Concomitant therapy	0 (0%)	16 (57%)	10 (50%)
Radiation therapy	0 (0%)	12 (43%)	5 (25%)
Targeted therapy	0 (0%)	3 (11%)	3 (15%)
Surgery	0 (0%)	0 (0%)	2 (10%)
Chemotherapy	0 (0%)	0 (0%)	2 (10%)
Other	0 (0%)	0 (0%)	2 (10%)
Clinician assessment of best response			
Complete response	0 (0%)	1 (4%)	0 (0%)
Partial response	0 (0%)	0 (0%)	2 (10%)
Stable disease	1 (50%)	6 (21%)	11 (55%)
Progressive disease	1 (50%)	19 (68%)	7 (35%)
Not assessed	0 (0%)	2 (7%)	0 (0%)
Reason for stopping			
Disease progression	2 (100%)	21 (75%)	17 (85%)
irAE	0 (0%)	7 (25%)	7 (35%)
Deceased	0 (0%)	2 (7%)	0 (0%)
Ongoing ICI	0 (0%)	0 (0%)	1 (5%)
irAEs	0 (0%)	12 (43%)	10 (50%)
Met toxicity criteria^a^	0 (0%)	7 (25%)	7 (35%)

*Note*: Data reported as median (interquartile range) or *n*/*N* (%). Percentages may add to more than 100% since patients can have multiple concomitant therapies or reasons for stopping.

Abbreviations: ICI, immune checkpoint inhibition; iRAE, immune‐related adverse event.

^a^ Met toxicity criteria were defined as unplanned clinic visits, emergency department visits, or unplanned hospital admissions that were at least possibly related to off‐label ICI.

### 3.6 Toxicity of off‐label ICI

**FIGURE 4:**
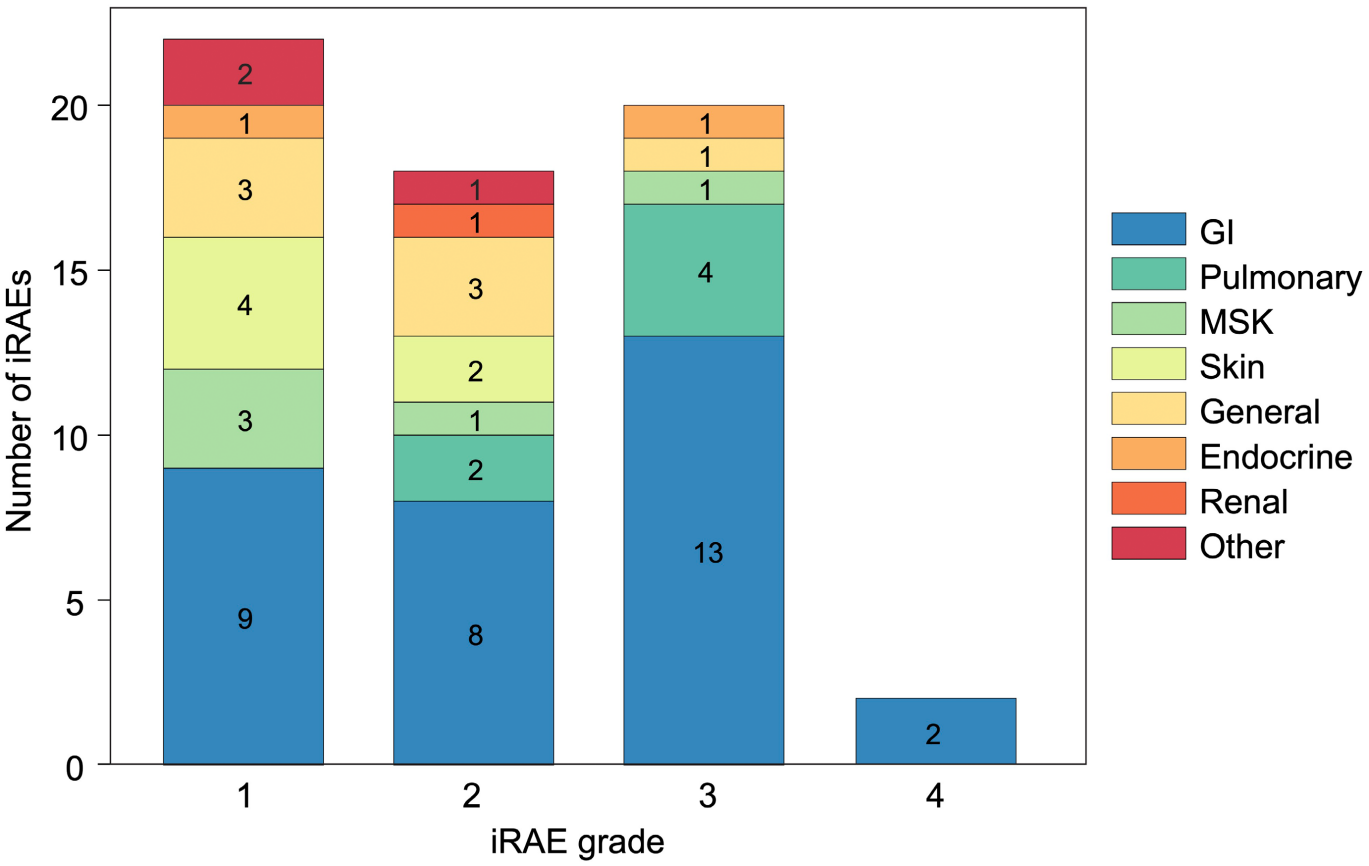
Number of immune‐related adverse events (irAEs) by severity graded according to Common Terminology for Criteria for Adverse Events (CTCAE) version 5.0 System Organ Class. iRAE data were extracted from the medical record and classified by reviewer interpretation. GI, gastrointestinal; MSK, musculoskeletal.

**FIGURE 5:**
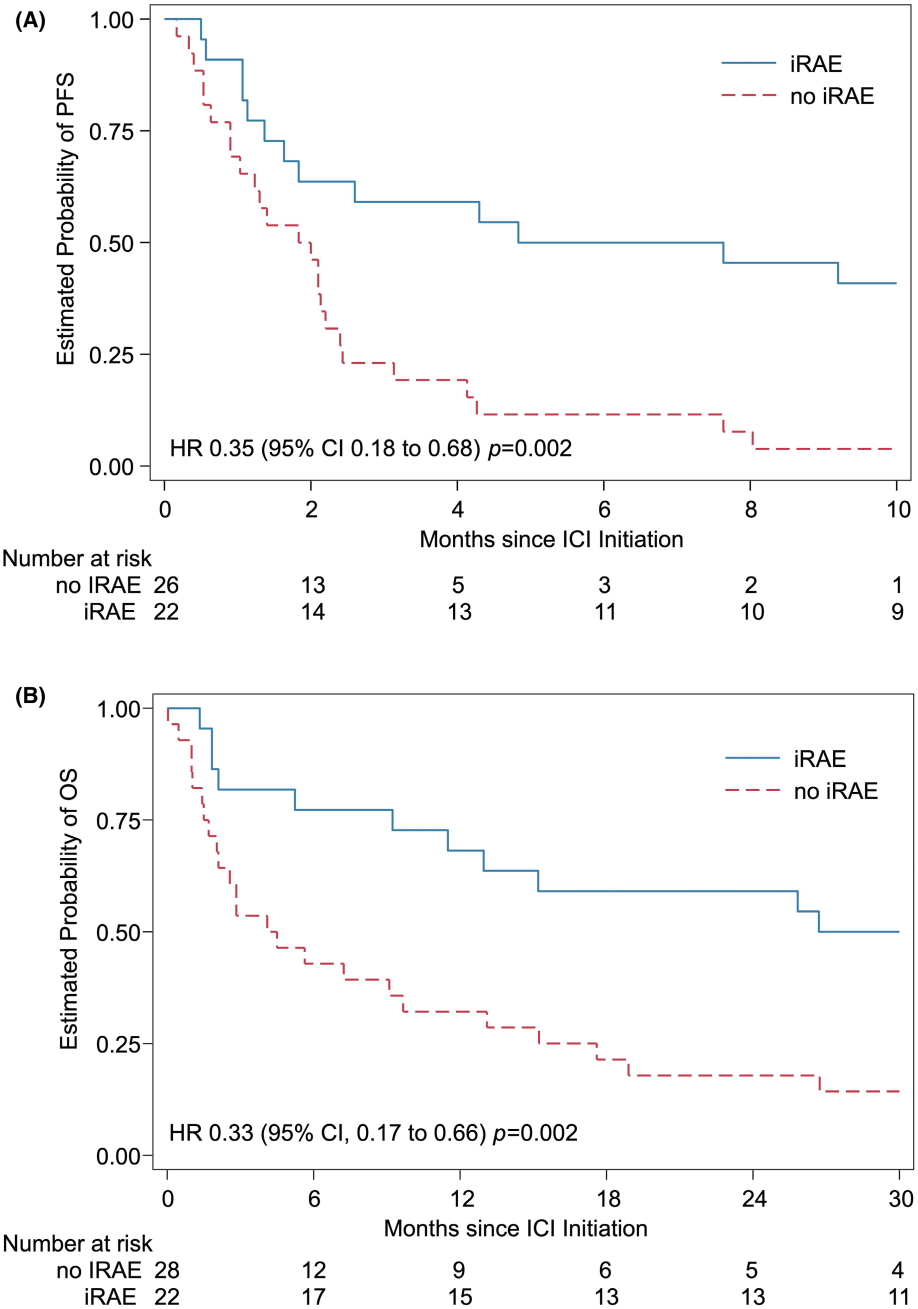
Estimated progression‐free survival (PFS) (A) and overall survival (OS) (B) according to immune‐related adverse event (irAE) occurrence.

There were 22 patients (44%) who experienced at least one irAE, with 12 of these patients experiencing multiple irAEs, resulting in 62 irAEs total (Figure 4). Ten unique patients experienced a grade 3 or higher irAE. Of the 22 patients who experienced an irAE, 11 were receiving pembrolizumab, nine were receiving nivolumab/ipilimumab, and two were receiving nivolumab. Gastrointestinal toxicities were the most common irAE overall (32/62, 52%). as well as the most common grade 3 or higher irAE (13/20, 65%). Of 20 grade 3 or higher irAEs, seven were hepatobiliary, and four were pancreatic. Both grade 4 irAEs were elevated AST. Overall, 14 patients (28%) had their ICI regimen interrupted by irAEs, with seven of these patients experiencing a grade 3 or higher irAE. All 14 of these patients met toxicity criteria (unplanned clinic visit, ED visit, or hospitalization), and 12 required immunosuppression with corticosteroids. Median (IQR) time from ICI initiation to irAE occurrence was 143 (35–420) days. Outcomes based on whether patients experienced an iRAE were evaluated. These data were adjusted for a priori defined covariates (disease category, ICI regimen), but did not account for cancer type, including natural history of disease, concomitant therapy, or timing of iRAE emergence. Experiencing an irAE was positively associated with survival outcome, with hazard ratios of 0.35 (95% CI, 0.18 to 0.68, *p* = 0.002) for estimated PFS and 0.33 (95% CI, 0.17 to 0.66; *p* = 0.002) for estimated OS (Figure 5). No deaths were directly attributable to iRAEs.

### 3.7 ICI rechallenge

Of six patients who were rechallenged with ICI after temporary discontinuation due to prior iRAE, three eventually experienced a recurrence of an irAE. One patient experienced irAE recurrence after their first dose of ICI rechallenge. This patient had initially developed a severe rash on nivolumab which recurred after a 4‐week break. The other two patients received multiple doses of ICI rechallenge prior to experiencing a recurrence of an irAE. The first patient had signs of pneumonitis while on nivolumab so the next dose was delayed 2 weeks. Subsequent rechallenge was successful, and the patient received nivolumab for more than a year without issues before colitis necessitated permanent discontinuation of ICI. The second patient was rechallenged with nivolumab 5 months after pneumonitis and received three doses before pneumonitis recurred. Two patients were successfully rechallenged with no irAE recurrence. The first had initially developed enteritis while on pembrolizumab, which was delayed for 2 months and then restarted without recurrence. The second had developed pneumonitis and pericarditis while on pembrolizumab which was delayed for 3 years before being restarted.

## 4 DISCUSSION

ICI has revolutionized cancer treatment and is now the standard of care for many types of cancer in adults. However, in the pediatric population, the role of ICI in cancer treatment remains uncertain. Nonetheless, ICI has shown promise in select groups of pediatric tumors, including those that are hypermutated or SMARCB1‐deficient.^35^, ^36^, ^37^, ^38^, ^39^, ^40^ Understanding the current landscape of off‐label ICI may inform future prescribing practices. Previously, our group described off‐label ICI use in 11 patients with CNS tumors.^24^ The present study extends follow‐up of these patients and includes additional patients with neuro‐oncology diagnoses, solid tumors, and hematologic malignancies. We found that the majority of patients in our cohort had metastatic disease and were treated in the relapsed/refractory setting, while only a minority had PD‐L1 and/or PD‐1 expression or TMB assessed prior to off‐label initiation. ICI also appeared to be well‐tolerated with a favorable toxicity profile, with most ICI discontinuation attributed due to disease progression rather than toxicity. Importantly, although most patients had disease progression after initiation of ICI, there were a few exceptional responders. Lastly, in this limited retrospective cohort, irAEs were associated with more favorable clinical outcomes.

The first instance of off‐label ICI use at our institution was in 2014. This coincided with the FDA granting accelerated approvals for pembrolizumab and nivolumab in patients with advanced melanoma who had progressed after ipilimumab treatment, following results from the KEYNOTE‐001 and CheckMate‐037 trials.^41^, ^42^ Subsequently, off‐label prescribing peaked in 2018. This was notable because in 2017–2018, the FDA approved ICI for several advanced pediatric cancers.^43^ Ipilimumab and nivolumab, both as monotherapy or in combination, were approved for the treatment of melanoma^12^, ^13^ and dMMR colorectal cancer,^14^, ^15^ while pembrolizumab was approved for the treatment of classical Hodgkin's lymphoma,^16^, ^17^ MSI‐H/dMMR solid tumors,^18^, ^19^ mediastinal large B‐cell lymphoma,^20^, ^21^ and as frontline therapy in Merkel cell carcinoma.^22^, ^23^ However, after 2018, there was a a decrease in off‐label ICI prescriptions. This trend might reflect increased on‐label prescribing due to FDA approvals that included pediatric indications, new clinical trials, improved understanding that ICI is ineffective for most pediatric cancers, or the COVID‐19 pandemic. During this time period, a number of clinical trials evaluating ICI in the pediatric setting reported their results. Two of the largest, KEYNOTE‐051 and Children's Oncology Group study ADVL1412, observed low antitumor activity of ICI in the majority of pediatric tumors.^27^, ^44^ These results may have discouraged subsequent prescribing of off‐label ICI due to less favorable perception of benefit. Over the last few years, new legislative initiatives, such as the RACE act, have sought to stimulate pediatric cancer drug development.^5^, ^45^ Additionally, there has been increasing support for lowering the age of eligibility in oncology clinical trials to include adolescents.^46^, ^47^ Initiatives such as these may influence future trial landscape and access to ICI.

While most patients did not respond to ICI, there were a few exceptional responders. One patient with melanoma had a complete response, which was not surprising since melanoma is known to be responsive to ICI.^12^, ^13^ In contrast, the durable partial responses observed in individual patients with high‐grade glioma and CNS nongerminatomous germ cell tumors were unexpected, since both diseases are typically incurable in recurrence and previous reports have not shown ICI to confer a clear survival advantage in pediatric patients with CNS tumors.^24^, ^48^ Our results add to others demonstrating the promise of ICI in neuro‐oncology.^24^

A significant number of patients treated with ICI experience irAEs. The incidence of irAEs is dependent on the ICI regimen, with anti‐CTLA‐4 regimens causing more toxicity than anti‐PD‐1 regimens, and dual‐agent ICI causing more toxicity than ICI monotherapy.^49^ In our cohort, 20% of patients experienced grade ≥3 irAE (classified according to CTCAE v5.0 by reviewer interpretation of clinic notes). In comparison, the KEYNOTE‐051 and ADVL1412 trials observed rates of 8% and 36% grade ≥3 treatment‐related adverse events, respectively.^27^, ^44^ Most of our grade ≥3 irAEs were gastrointestinal, more specifically hepatobiliary, a finding that has also been observed in previous studies.^50^, ^51^ We also observed improved outcomes in patients who experienced irAEs. Our study adds to several others that have observed a positive association between irAE occurrence and ICI efficacy.^12^, ^52^, ^53^, ^54^, ^55^, ^56^, ^57^, ^58^, ^59^, ^60^, ^61^, ^62^, ^63^, ^64^, ^65^, ^66^

Although the association between mutational burden and ICI response is well‐established, the role of PD‐L1 expression in predicting response to ICI is less clear. Several studies have observed improved survival in patients with tumors that have high PD‐L1 expression,^67^, ^68^, ^69^ whereas others question the robustness of PD‐L1 expression as a predictive biomarker.^70^, ^71^ Most pediatric tumors express low levels of PD‐1, PD‐L1, and PD‐L2.^72^, ^73^, ^74^ Nonetheless, it is clear that some tumors lacking PD‐L1 can still respond to ICI. In the present study, most patients did not undergo PD‐L1 staining or TMB assessment prior to ICI initiation. Reasons for this are multifactorial and may include the finding that most pediatric tumors have low TMB,^75^ controversy surrounding the generalizability of TMB as a predictive biomarker of ICI response,^76^, ^77^, ^78^ and availability of a reliable assay during the time period of our study. It is clear that ICI has efficacy in treating a subset of hypermutated pediatric tumors.^14^, ^15^, ^18^, ^19^ Ongoing studies are needed to determine the therapeutic utility of assessing TMB as a predictive biomarker in the pediatric setting.

The two major strengths of this study are that it is the largest and most inclusive study to date describing patterns of off‐label ICI use in any setting, and that it reflects prescribing patterns at a large tertiary pediatric oncology referral center with diversity in patients and providers. Our study is, however, not without limitations. The study is retrospective, representative of prescribing bias, and therefore not readily generalizable to all pediatric patients. Rationale for prescribing was not captured. Clinical assessments of best response were derived from clinical notes rather than image‐guided assessments. Ideally, best response would have included radiographic evaluations by pediatric radiologists, but this was beyond the scope of this study. Additionally, response assessment did not consider other factors that may cause disease stabilization, such as the natural history of disease and concomitant therapies. CTCAE classification of iRAEs was derived from the medical record and may be subject to interpretation. Finally, we report on a single‐institution experience that may not be generalizable to other settings. Observational studies with prospectively collected data, such as the ongoing French study, SACHA,^79^ are needed to clarify the patterns, safety, and efficacy of off‐label ICI use.

In conclusion, this study describes patterns of off‐label ICI prescribing at a large pediatric cancer center and reaffirms the safety of ICI in the pediatric setting, with antitumor activity in rare subgroups of patients. These results may better inform shared decision‐making regarding off‐label ICI usage and reinforce the need for additional research to identify patients likely to benefit from ICI.

## AUTHOR CONTRIBUTIONS

**Ajami Gikandi:** Data curation (equal); formal analysis (equal); investigation (lead); writing – original draft (equal). **Susan N. Chi:** Resources (equal); writing – review and editing (equal). **Kee Kiat Yeo:** Resources (equal); writing – review and editing (equal). **Allison F. O'Neill:** Resources (equal); writing – review and editing (equal). **David S. Shulman:** Conceptualization (supporting); methodology (equal); writing – review and editing (equal). **Steven G. DuBois:** Conceptualization (supporting); supervision (equal); validation (equal); writing – review and editing (equal). **Natalie B. Collins:** Conceptualization (equal); data curation (equal); formal analysis (equal); investigation (supporting); methodology (equal); writing – original draft (equal).

## FUNDING INFORMATION

The author(s) received no financial support for the research, authorship, and/or publication of this article.

## CONFLICT OF INTEREST STATEMENT

Steven G. DuBois has received consulting fees from Amgen, Bayer, and Jazz and travel expenses from Loxo Oncology, Roche, and Salarius. David S. Shulman has received consulting fees from Boehringer Ingelheim and serves on the scientific advisory board of Merlin Biotech. Allison F. O'Neill has received consulting fees from Fennec Pharmaceuticals and Skyline Therapeutics.

## Data Availability

Data available on request due to privacy/ethical restrictions.
